# Seroprevalence of influenza A and B viruses among unvaccinated children in the United Arab Emirates: a cross-sectional study

**DOI:** 10.1186/s13104-017-2720-8

**Published:** 2017-08-10

**Authors:** Ahmed R. Alsuwaidi, Lolowa A. Al-Mekaini, Salwa M. Kamal, Hassib Narchi, Abdul-Kader Souid

**Affiliations:** 1 Department of Pediatrics, United Arab Emirates University, P.O. Box 17666, Al Ain, UAE; 2 Ambulatory Healthcare Services, Abu Dhabi Health Services Company (SEHA), Abu Dhabi, UAE

**Keywords:** Communicable diseases, Influenza, Influenza vaccine, Serosurvey

## Abstract

**Background:**

Young children are at increased risk of severe influenza disease and, thus, are good candidates for receiving annual vaccination. Nevertheless, the influenza vaccine is infrequently given to children in our region. The primary objectives of this study are to monitor the serologic immunities against influenza A and B viruses, and provide pediatric data that support the need for influenza vaccination in the community.

**Methods:**

Influenza A and B virus-specific IgG antibodies were measured in 294 children (median age 4.1 years; range 1.9–12.5 years) between July 2014 and September 2015.

**Results:**

The percentage of children who were seropositive for influenza A IgG was 15.8%, equivocal 7.4%, and negative 76.8%. The corresponding values for influenza B IgG were 31.3, 9.6, and 59.1%, respectively. There was a higher seropositivity rate for influenza B than for influenza A in all age groups. The percentage of children who were seropositive for either influenza A or B IgG was 27.9% and for both was only 2.7%.

**Conclusions:**

Most of the studied children are serologically naïve and, thus, are likely to acquire primary influenza disease. A national policy that endorses childhood influenza vaccination is highly advisable.

## Background

Influenza A and B viruses frequently infect children, with a reported seasonal attack rate of about 18% in children younger than 3 years [1]. Children also contribute efficiently to the spread of these viruses in the community [1]. Nevertheless, the influenza vaccine is infrequently given to infants and toddlers, who are usually serologically naïve and prone to more severe disease [2]. In addition, children who have chronic health problems (e.g., asthma, cardiovascular disease, sickle cell anemia, or premature birth) are also at increased risk of serious complications from influenza virus infection [3]. Therefore, controlling the pediatric influenza disease should be considered a public health priority.

Annual vaccination is an available intervention that may limit the spread of influenza A and B viruses [4]. The efficacy of this approach is highly dependent on matching the vaccine strains with those circulating in the region [4]. Serosurveys are needed to support campaigns that provide annual vaccination.

The Health Authority of Abu Dhabi in the United Arab Emirates (UAE) emphasizes the importance of annual influenza vaccine as an effective prevention strategy in the community with priority given to healthcare workers, pregnant women, Hajj and Umrah pilgrims, smokers, adults more than 65 years and other high-risk groups including adults and children with diabetes, asthma, kidney failure, chronic cardiovascular disease and chronic liver disease [5]. The vaccine is also available for people who request the vaccine to protect themselves. There is, however, no policy for routine seasonal influenza vaccination in young children who do not belong to those high-risk groups. In 2015, most of the notified influenza cases in the emirate of Abu Dhabi were children (35% in 0–4 years, 15% in 5–9 years, 5% in 10–14 years, and 45% in ≥15 years). Most of the infections were due to influenza B and occurred in January–April and October–December [5].

Here, we report the results of the serosurvey of influenza A and B virus-specific IgG antibodies in Emirati children. The studied sera were collected in governmental ambulatory pediatric clinics in Abu Dhabi.

## Patients and methods

This cross-sectional study involved a non-selected cohort of 294 Emirati children who attended the Well-Child-Care Program of Ambulatory Healthcare Services (Al-Ain, Abu Dhabi) between July 2014 and September 2015 [6]. Children (23 months to 12 years of age) who presented to these services for routine care were enrolled in this study if the parents consented and they had no documented acute or chronic illnesses or regular medications. Their medical records were reviewed for influenza immunization history.

Blood was collected and processed as part of a study to measure the immune responses to vaccine preventable diseases including influenza A and B viruses [6]. Enzyme-linked immunosorbent assay (ELISA) was used to measure influenza A and B virus-specific Immunoglobulin G (IgG) antibody titers in the sera of participating children using RIDASCREEN^®^ IgG (R-Biopharm, Darmstadt, Germany). All tests were performed and interpreted according to the manufacturers’ instructions in the single central reference laboratory of the Ambulatory Healthcare Services (Abu Dhabi). Results were expressed based on a standard curve provided with the assay. In the influenza A IgG ELISA, samples were considered positive if the antibody concentration was >5 U/mL, a range of 4–5 U/mL was considered equivocal, and values <4 U/mL were interpreted as negative. The corresponding cut-off values for influenza B IgG were >8 U/mL (positive), 5–8 U/mL (equivocal), and <5 U/mL (negative). Equivocal and negative results were confirmed by repeated testing. Positive serology results in this study reflected the immune response to the circulating viruses, which typically last up to 6 months [6].

### Statistical analysis

The data were analyzed with the SPSS statistical package (version 20). Statistical significance was defined by a 2-sided *p* value <0.05.

## Results

The mean ± SD age (years) was 4.6 ± 2.2 (median 4.1, range 1.9–12.5). Only one child (7.8 years) had documentation of receiving the influenza vaccine once 5 years prior to the study sample collection; her serology was negative for influenza A virus and positive for influenza B virus.

**Fig. 1:**
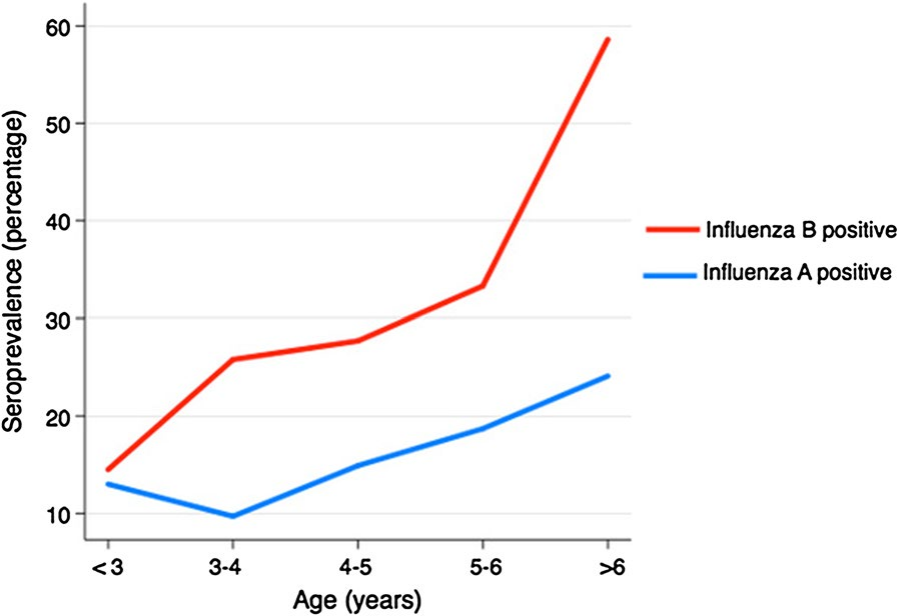
Serology by age. The percentages of children with positive serology for either influenza A or B viruses by age are shown

**Fig. 2:**
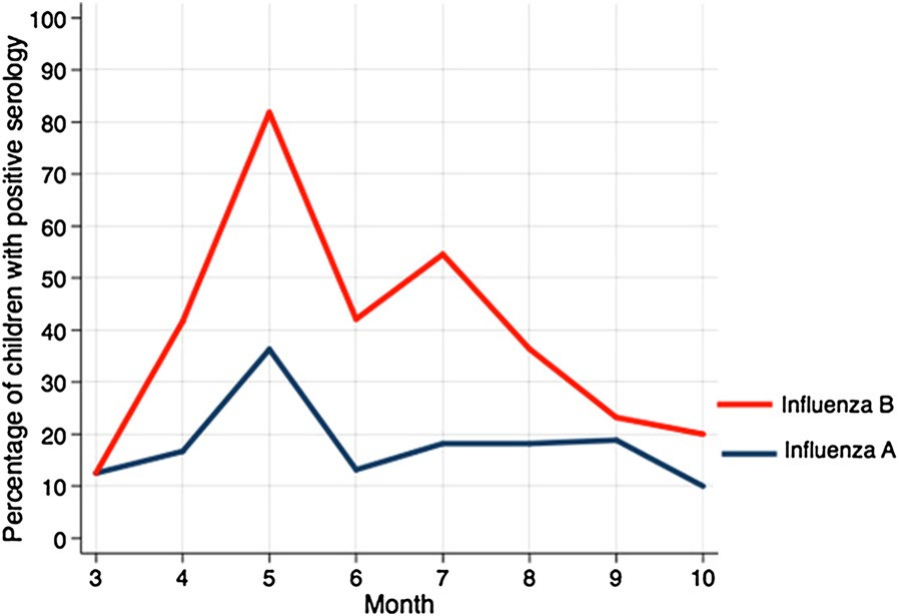
Serology by month of testing. The percentages of children with positive influenza serology by month of testing are shown

The seroprevalence of influenza A and B IgG as function of age is shown in Table 1 and Fig. 1. The positive serology rate by month of testing is shown in Fig. 2. Overall, the percentage of children who were seropositive for influenza A IgG was 15.8%, equivocal 7.4%, and negative 76.8%. The corresponding values for influenza B IgG were 31.3, 9.6, and 59.1%, respectively. There was a higher seropositivity rate for influenza B than for influenza A in all age groups (Table 1; Fig. 1). The percentage of children who were seropositive for either influenza A or B IgG was 27.9% and for both was only 2.7%.

**Table 1:** Seroprevalence of influenza A and B virus-specific IgG antibodies as function of age

Age (years)	Influenza A			Influenza B		
	Positive	Equivocal	Negative	Positive	Equivocal	Negative
<3	13.0 (9)	5.8 (4)	81.1 (56)	14.5 (10)	5.8 (4)	79.7 (55)
3–4	9.7 (6)	6.4 (4)	83.9 (52)	25.8 (16)	4.8 (3)	69.3 (43)
4–5	14.9 (7)	8.5 (4)	76.6 (36)	27.7 (13)	10.6 (5)	61.7 (29)
5–6	18.7 (9)	6.2 (3)	75.0 (36)	33.3 (16)	14.6 (7)	52.1 (25)
6–12.5	24.1 (14)	10.3 (6)	65.5 (38)	58.6 (34)	13.8 (8)	27.6 (16)
All	15.8 (45)	7.4 (21)	76.7 (218)	31.3 (89)	9.5 (27)	59.1 (168)

Values are percent (n). Serologic studies were missing in some patients due to inadequate serum

## Discussion

This cross-sectional study investigated the prevalence of influenza A and B virus-specific IgG antibodies among Emirati children in our community. Antibodies (positive + equivocal) to influenza B virus were more prevalent than those to influenza A virus (40.8% vs. 23.2%), Table 1. These results reflect the circulating virus during the study period [5]. The seropositivity to influenza A and B was especially high in March (Fig. 2).

The positive serology results obtained in our study probably reflect the immune response to the circulating viruses, which typically last up to 6 months. In UAE, vaccination against seasonal influenza is usually given only to children who have chronic health problems; these children are not included in this study.

In one study from Germany, 40% of children <4 years were serologically naïve to influenza A virus, and 80% of children <6 years were serologically naïve to influenza B virus [7]. The authors have commented that many of these young children are expected to acquire primary infections [7]. In another study from the Netherlands, the proportion of children with detectable antibodies against influenza A and B viruses gradually increased with age until 6 years; thereafter, they all had antibodies to at least one virus [8]. As shown in Table 2, seropositivity prevalence for influenza A virus is much less in our study than that in those from Germany or the Netherlands, while the seropositivity prevalence for influenza B virus is somewhat similar for the three populations (Table 2). Some possible reasons for the observed differences include the fact that the circulation of seasonal influenza viruses varies across geographic regions and with seasons. Another possible explanation may be the difference in sensitivity of the ELISA assays used in our study (R-Biopharm) in comparison to the ELISA used in the study from Germany (IBL International for influenza A and Euroimmun for influenza B); and also the study from the Netherlands, which had used instead the hemagglutination-inhibition assay. Commercial ELISA tests measure antibodies that bind to influenza virions while hemagglutination-inhibition assays measure functional antibodies (inhibition of hemagglutination). While ELISA tests have the advantage of completion within a few hours and can be fully automated for larger sample size, they lack, however, the ability to discriminate between antibodies against various antigenically distinct influenza A and B subtypes. The hemagglutination-inhibition and microneutralization assays, on the other hand, can detect strain-specific serum antibodies. This is important for monitoring antigenic changes during influenza A and B epidemics [9]. Future studies should consider assays that address influenza A subtypes and utilize standardized protocols and antibody standards to improve reproducibility of the obtained results. Another limitation of the current report, which needs to be addressed in future studies, is the unavailability of data on influenza related mortality.

**Table 2:** Seropositivity prevalences for influenza A and B virus-specific IgG antibodies among children in various nations

Age (years)	Influenza A virus			Influenza B virus		
	Germany 2008–2011 (%) [7]	Netherlands 2006–2007 (%) [8]	This study 2014–2015 (%)	Germany 2008–2010 (%) [7]	Netherlands 2006–2007 (%) [8]	This study 2014–2015 (%)
≤4	59.6	64^a^	13.0	10.7	27^a^	22.4
5–8	92.9	99^b^	25.3	30.4	72^c^	55.2
9–12	99.5	–	35.7	59.8	–	75.0

^a^Average prevalences in children 1–4 years old

^b^6–7 years old

^c^7 years old, retrieved from the text and Figure 3 of Ref. [8]

## Conclusions

The majority of the studied children, especially the toddlers, are seronegative for both influenza A and B viruses. Thus, these children are likely to acquire primary influenza disease and may benefit from annual vaccination. The data also support the need for a national influenza vaccination strategy that includes all children. The influenza vaccine should target common circulating strains in our community.

## Abbreviations

UAE: United Arab Emirates
ELISA: enzyme-linked immunosorbent assay
IgG: immunoglobulin G
